# Associations between cognitive function and lifestyle factors in healthy Japanese middle-aged and older adults: A cross-sectional study

**DOI:** 10.1371/journal.pone.0348439

**Published:** 2026-05-04

**Authors:** Koki Tsuda, Kotatsu Bito, Masanobu Hibi, Takahiro Ono, Hiroshi Maruyama

**Affiliations:** 1 Digital Business Creation, Life Care Business Development, Kao Corporation, Chuo-ku, Tokyo, Japan; 2 Analytical Science Research Laboratories, Kao Corporation, Sumida-ku, Tokyo, Japan; 3 Human Health Care Product Research Laboratories, Kao Corporation, Sumida-ku, Tokyo, Japan; 4 Ueno-Asagao Clinic, Taito-ku, Tokyo, Japan; 5 Preferred Networks Inc., Chiyoda-ku, Tokyo, Japan; 6 Research into Artifacts, Center for Engineering, The University of Tokyo, Bunkyo-ku, Tokyo, Japan; Japanese Academy of Health and Practice, JAPAN

## Abstract

The objective of this study is to comprehensively investigate associations between cognitive function and lifestyle factors. We analyzed data from a cross-sectional study of Japanese adults that included approximately 1,800 variables (e.g., physical characteristics, body composition, and lifestyle habits) and cognitive function assessed using CNS Vital Signs. For participants aged 40 years or older (*n* = 710), we performed partial correlation analysis and analysis of covariance adjusted for sex, age, and years of education. Given the large number of variables, we controlled the false discovery rate within predefined data types (real, positive, ordered categorical, categorical) using the Benjamini–Hochberg procedure. We adjusted *p*-values to *q*-values and identified variables with *q* < 0.1 for exploratory purposes. In total, 28 variables met this criterion, with particularly prominent associations for gait characteristics, vascular function, grip strength, and oral conditions, whereas blood components and other general biomarkers did not meet the threshold. These exploratory findings identify candidate correlates of cognitive function in this relatively healthy, primarily urban Japanese cohort and require confirmation in independent longitudinal studies.

## Introduction

Population aging is advancing in many countries, and cognitive decline among older adults has become a major public health concern [1]. By 2050, the number of older adults living with dementia is globally projected to reach approximately 150 million, underscoring the urgent need for multifaceted strategies to address this challenge [2]. Cognitive decline is known to trigger a variety of health problems, including physical frailty, and to significantly impair quality of life [3]. In addition, dementia imposes substantial medical costs and informal caregiving burdens, potentially leading to severe economic impacts on society [4].

To prevent the onset of dementia, increasing attention has been directed toward early at-risk or prodromal stages such as mild cognitive impairment (MCI) and subjective cognitive decline (SCD) [5,6]. MCI is characterized by mild cognitive decline that does not interfere with daily life, whereas SCD refers to a state in which objective cognitive impairment is not detectable, yet individuals subjectively perceive a decline. People with MCI or SCD are known to be at an elevated risk of developing dementia [7,8]. However, it is also well established that appropriate interventions, such as lifestyle modifications and structured programs, can delay the progression of cognitive decline [9,10].

Previous studies have reported that regular physical exercise [11,12] and dietary interventions, such as adherence to a Mediterranean diet [13,14], may slow cognitive decline. Psychological well-being also plays an important role. In particular, stress management strategies, including meditation and mindfulness, have been suggested to reduce stress and enhance cognitive performance [15]. Furthermore, active participation in social activities has been shown to provide cognitive stimulation that may help maintain brain function [16,17]. Taken together, these findings underscore the importance of encouraging the early detection of cognitive decline and motivating individuals to engage in preventive lifestyle interventions.

A range of methods has been developed to assess cognitive decline, including cognitive tests such as the Mini-Mental State Examination [18] and Montreal Cognitive Assessment [19], detection of biomarkers such as amyloid-β and tau [20], and neuroimaging using positron emission tomography [21]. However, although these methods are appropriate for patients with apparent symptoms, they are not well suited for continuous, long-term monitoring of asymptomatic individuals in terms of feasibility and cost [22]. Moreover, biomarker assessment typically requires cerebrospinal fluid sampling via lumbar puncture, which is invasive [22], and brain imaging requires expensive equipment and infrastructure [23]. For these reasons, there is a growing need for more accessible, cost-effective approaches that leverage data on lifestyle factors to detect early and subtle cognitive changes at the population level [24].

In our previous work, we collected comprehensive cross-sectional data on lifestyle factors from Japanese adults [25]. This dataset includes approximately 1,800 variables encompassing physical characteristics, body composition measures, and lifestyle habits, all measured in the same participants. Importantly, it also includes results from cognitive assessments performed using CNS Vital Signs (CNSVS), a convenient tool for evaluating multiple cognitive domains, including memory, attention, and executive function [26]. Building on prior findings, the present study investigates associations between cognitive function and a broad range of lifestyle factors and aims to provide evidence-based recommendations to help maintain cognitive health and promote healthy aging.

## Materials and methods

### Study design and participants

This study was performed as exploratory analysis using data from a previously reported single-center cross-sectional observational study [25]. The protocol of the study is briefly summarized below.

The study was approved by the Institutional Review Boards (IRBs) of Kao Corporation (Tokyo, Japan; approval number K0023-2108) in October 2021. Thereafter, for data analysis, an ethics review application was submitted to the IRBs of Preferred Networks Inc. (Tokyo, Japan; approval number ET22110047) and approved in December 2022. Participants were consecutively recruited from 19 October 2021 to 4 February 2022. Recruitment was conducted through a website operated by TES Holdings (Tokyo, Japan). The inclusion criteria were: (1) Japanese men and women aged 20 years or older, (2) ability to complete questionnaires and surveys, and (3) provision of written informed consent after understanding the study procedures. The exclusion criteria were: (1) hospitalization for severe diseases (e.g., diabetes, hypertension, arteriosclerosis, heart disease, malignant tumor, Alzheimer’s disease), (2) inability to attend the clinic independently, (3) diagnosis or suspected diagnosis of dementia, (4) symptoms suggestive of COVID-19 infection, (5) use of a cardiac pacemaker, (6) current pregnancy or possible pregnancy, and (7) any other condition deemed inappropriate for participation by the principal investigator.

All participants provided written informed consent, which detailed the data elements to be used and included consent for use of anonymized data. Participants visited Ueno-Asagao Clinic (Tokyo, Japan) twice at one-week intervals. All measurements were performed by trained research coordinators and physicians following standardized operating procedures. The study was conducted in accordance with the Strengthening the Reporting of Observational Studies in Epidemiology (STROBE) guidelines. The study protocol was prospectively registered at the University Hospital Medical Information Network (UMIN000045746) on 14 October 2021.

### Measurement items

#### Cognitive function.

Cognitive function was assessed using the standardized Neurocognition Index (NCI) score obtained from the CNSVS battery [26]. CNSVS is a computerized neurocognitive screening tool that evaluates multiple cognitive domains and is widely used for the assessment of cognitive function. The standardized scores are normalized for each age group based on the CNSVS normative database, with a mean of 100. Among these scores, the NCI score represents a global measure of neurocognitive function, calculated as the mean of composite memory, psychomotor speed, reaction time, complex attention, and cognitive flexibility. Consistent with our objective of comprehensively examining the relationship between overall cognitive function and lifestyle factors, we employed the NCI score as a global indicator of cognitive function rather than scores for specific cognitive domains.

#### Physical characteristics, body composition, and lifestyle habits.

Height was measured using a standard stadiometer, weight was measured using a body composition analyzer (InBody 770K, InBody Corp., Seoul, Korea), and body mass index (BMI) was calculated. Blood pressure (systolic and diastolic), cardio-ankle vascular index (CAVI), ankle-brachial index (ABI), and other vascular parameters were measured using a VS-2500 Vascular Screening System (Fukuda Denshi Co., Ltd., Tokyo, Japan). Grip strength was measured twice with a Smedley-type dynamometer (GRIP-D, Takei Scientific Instruments Co., Ltd., Tokyo, Japan), and the mean value was recorded.

Gait parameters including walking speed, stride length, step width, foot angle, stance and swing phases, and plantar pressure distribution were measured on a 6.4-m walkway equipped with a sheet-type foot pressure sensor (Anima Corp., Tokyo, Japan); the mean of four trials was used for analysis. Daily walking speed and step counts were recorded continuously for 14 days using a tri-axial accelerometer (HW-100, Kao Corporation, Tokyo, Japan) and a smartphone-based gait analysis application (Chami, InfoDeliver Co., Ltd., Tokyo, Japan).

Information on medical history, medication use, and lifestyle factors, including diet, sleep, psychological health, stress, excretory function, and menopausal status, was obtained through physician or coordinator interviews and a series of validated questionnaires: the brief-type self-administered diet history questionnaire (BDHQ) [27], International Physical Activity Questionnaire (IPAQ) [28,29], Questionnaire for Medical Checkup of Old-Old (QMCOO) [30], dietary habits questionnaire [31], Athens Insomnia Scale [32,33], Oguri–Shirakawa–Azumi Sleep Inventory MA version (OSA-MA) [34], Berlin Questionnaire [35], World Health Organization–Five Well-Being Index (WHO-5) [36], Brief Job Stress Questionnaire (BJSQ) [37], Chalder Fatigue Scale [38], Center for Epidemiologic Studies Depression Scale (CES-D) [39,40], Fatigue Feelings Questionnaire [41], Sun Exposure Questionnaire [42], Ten-Item Personality Inventory (TIPI-J) [43,44], Oxford Happiness Questionnaire [45], Overactive Bladder Symptom Score (OABSS) [46], International Consultation on Incontinence Questionnaire–Urinary Incontinence Short Form (ICIQ-UI SF) [47], Fecal Incontinence Quality of Life (FIQL) scale [48], Neurogenic Bowel Dysfunction (NBD) score [49], Kupperman Menopausal Index [50], modified Menstrual Distress Questionnaire (mMDQ) [51,52], climacteric and senescence scores [53], and, for participants within 3 years postpartum, the Edinburgh Postnatal Depression Scale (EPDS) [54]. Oral health indicators, including tooth and gum condition, halitosis, and oral dryness were assessed using a rating questionnaire developed specifically for this study.

In addition, various physiological and biochemical parameters were measured using the methods described in protocol [25] and included in the analyses. Assessments included physical performance tests, laboratory analysis of blood, urine, and saliva, an oral glucose tolerance test, liquid chromatography–tandem mass spectrometry analysis of chiral amino acids, lipid mediators, vitamin D metabolites, and polyamines, assessment of hair loss, analysis of hand surface characteristics, analysis of stratum corneum lipids and sebum, analysis of body odor, skin surface spectroscopy, and analysis of RNA in skin surface lipids (SSLs).

### Statistical analyses

#### Analysis plan.

This study was an exploratory analysis aimed at hypothesis generation, and a prespecified analysis plan was established before the analyses were conducted. The primary outcome was the CNSVS NCI score. The covariates were sex, age, and years of education. The statistical methods were partial correlation analysis and analysis of covariance (ANCOVA). Multiple testing correction using the Benjamini-Hochberg procedure [55] was also prespecified, with an exploratory screening threshold of *q* < 0.1 to flag candidate associations in this high-dimensional setting. The interpretations of associations for variables that passed the exploratory false discovery rate (FDR) threshold, as well as any additional visualizations (e.g., scatter plots or box plots), are considered exploratory.

#### Data and analytical overview.

The data snapshot used for the analyses were downloaded on 23 July 2024. Only records that did not contain any personally identifiable information were used for analyses, and investigators had no access to such information.

The dataset included approximately 1,800 variables describing anthropometric measures, body composition, and lifestyle factors (see Measurement items). Because sex, age, and years of education are widely recognized as major determinants of cognitive performance [56–58], we calculated partial correlation coefficients and performed ANCOVA to control for their potential confounding effects. To address the issue of multiple comparisons, *p*-values were adjusted using the Benjamini–Hochberg procedure to yield *q*-values, thereby controlling FDR. As prespecified in the analysis plan, we used *q* < 0.1 (FDR 10%) as an exploratory screening threshold in this high-dimensional setting. This threshold was chosen to prioritize sensitivity for discovery while controlling the expected proportion of false discoveries, consistent with prior exploratory screening studies [59,60]. This threshold is not intended to provide confirmatory evidence. We recognize that it may include false positives and therefore should be replicated in independent cohorts and prospective study designs. All statistical analyses were conducted in Python (v3.9.20) using the pingouin (v0.5.5), statsmodels (v0.14.4), and SciPy (v1.13.1) packages.

Each variable was assumed to be one of four data types: real (continuous real-valued), positive (continuous and strictly positive), ordered categorical (ordinal), or categorical (nominal). An overview of the variables and their summary statistics is available in a separate paper [61]. Appropriate methods for handling missing values and for conducting statistical tests were applied accordingly. All code used in this study is provided in the supplementary materials (S1 Code and S2 Code).

#### Treatment of missing data.

The dataset contained missing values in approximately 0.2%–57% of variables [61]. Appropriate handling of missing data is essential for valid statistical inference [62]. However, given the large number of lifestyle-related variables, it was not feasible to apply tailored imputation methods for each variable while accounting for inter-variable relationships. Therefore, we adopted a complete-case analysis approach, excluding participants with missing data on the variables included in each analysis. We acknowledge that this approach may introduce bias into the estimated parameters.

#### Analysis of real, positive, and ordered categorical variables.

For variables classified as the real, positive, or ordered categorical type, we calculated partial Spearman correlation coefficients between each variable and the NCI score, adjusting for sex, age, and years of education, and obtained *p*-values. Only participants with complete data for the variables were included in this analysis. Cognitive subdomain scores other than the NCI score were excluded because they were expected to be highly correlated with the NCI score. After applying FDR correction within each data type, variables with *q* < 0.1 were identified for further interpretation.

#### Analysis of categorical variables.

For the categorical type variables, ANCOVA was performed with the NCI score as the dependent variable and sex, age, and years of education as covariates. Only participants with complete data for the variables were included in this analysis. Continuous variables were standardized, and categorical variables were converted into dummy variables before inclusion in the model. As a preliminary assumption check, interaction terms between each categorical variable and the covariates were included in the model to assess homogeneity of regression slopes. Variables with non-significant interaction terms (*p* > 0.05) were considered to satisfy this assumption. Homogeneity of variance was further assessed using Levene’s tests (*p* > 0.05), and variables that did not meet these assumptions were excluded from the subsequent ANCOVA. After applying FDR correction, variables with *q* < 0.1 were identified for further interpretation.

## Results

### Participants

**Fig 1:**
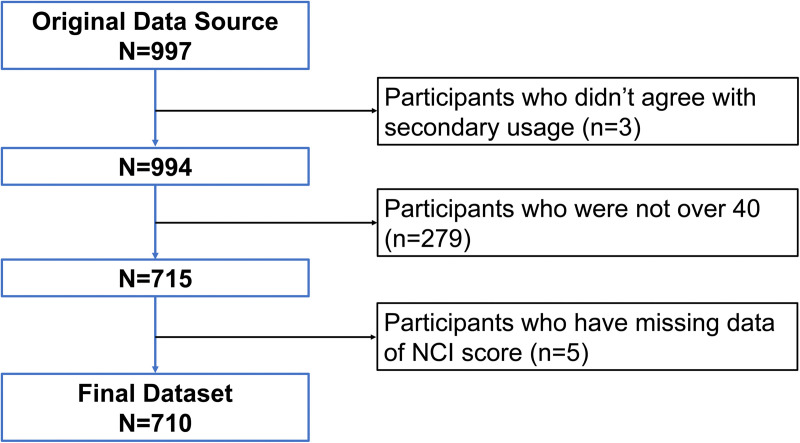
Participant selection flow. A flow diagram showing the inclusion and exclusion process of study participants. Among the 997 initially recruited Japanese adults, three individuals who did not provide consent for secondary use of data, 279 participants younger than 40 years, and five participants with missing NCI scores were excluded, resulting in 710 participants included in the final analysis.

The dataset included 997 Japanese adults. After excluding three individuals who did not provide consent for secondary use of data, 994 participants remained. Among these, 715 participants aged 40 years or older, who constituted the target population for examining potential age-related cognitive decline, were selected. After excluding five participants with missing NCI scores, a total of 710 participants were included in the final analysis. The participant selection flow is illustrated in Fig 1. Summary statistics of the demographic and clinical characteristics of the analyzed participants are presented in Table 1.

**Table 1:** Detailed demographic, lifestyle, and cognitive characteristics of participants included in the final analysis (*n* = 710).

Variables	*n*	%
**Sex**		
Male	349	49.2
Female	361	50.8
**Age (59.3 ± 11.2 [40–79])**		
40 ≤ to < 50	183	25.8
50 ≤ to < 60	164	23.1
60 ≤ to < 70	161	22.7
70 ≤ to < 80	202	28.5
**BMI (22.8 ± 3.7 [13.5–41.0])**		
Underweight (< 18.5)	79	11.1
Normal range (18.5 ≤ to < 25)	459	64.6
Pre-obese (25 ≤ to < 30)	142	20.0
Obese class I (30 ≤ to < 35)	26	3.7
Obese class II (35 ≤ to < 40)	2	0.3
Obese class III (≥ 40)	2	0.3
**Years of education (15.0 ± 2.3 [6–30])**		
≤ 9	15	2.1
9 <to ≤ 12	132	18.6
12 <to ≤ 16	480	67.6
16 <to ≤ 18	57	8.0
18 <to ≤ 21	20	2.8
> 21	6	0.8
**Smoking status**		
Never smoker	411	57.9
Former smoker	206	29.0
Current smoker (1–14 cigarettes per day)	50	7.0
Current smoker (15–29 cigarettes per day)	40	5.6
Current smoker (≥ 30 cigarettes per day)	3	0.4
**Drinking frequency**		
Every day	148	20.8
Sometimes	292	41.1
Seldom	270	38.0
**Exercise frequency**		
Almost never	277	39.0
Once per month	17	2.4
Two or three times per month	31	4.4
Once per week	77	10.8
Two to four times per week	161	22.7
Five or six times per week	88	12.4
Every day	59	8.3
**NCI score (102.4 ± 10.7 [48–124])**		
–	710	100.0

This table shows the distribution of participants’ demographic and lifestyle characteristics. Continuous variables are expressed as mean ± standard deviation [range]. “Years of education” represents the total number of years of formal schooling completed, including primary, secondary, higher, and postgraduate education. “Drinking frequency” categories were derived from the standardized Japanese health-check questionnaire administered at the time of data collection [63]. The questionnaire provided qualitative categories (Every day/Sometimes/Seldom) without quantitative frequency thresholds (e.g., times/week or times/month). BMI, body mass index [kg/m^2^]; NCI, Neurocognition Index.

The final dataset included 349 men (49.2%) and 361 women (50.8%), with a mean age of 59.3 ± 11.2 years, mean BMI of 22.8 ± 3.7 kg/m^2^, and mean years of education of 15.0 ± 2.3. Most participants were either never smokers (*n* = 411, 57.9%) or former smokers (*n* = 206, 29.0%). Regarding alcohol consumption, the majority reported drinking occasionally (*n* = 292, 41.1%) or rarely (*n* = 270, 38.0%). With respect to exercise habits, most participants reported either little to no regular exercise (*n* = 277, 39.0%) or exercising two to four times per week (*n* = 161, 22.7%). The mean NCI score was 102.4 ± 10.7.

### Analyses of associations with cognitive function

**Fig 2:**
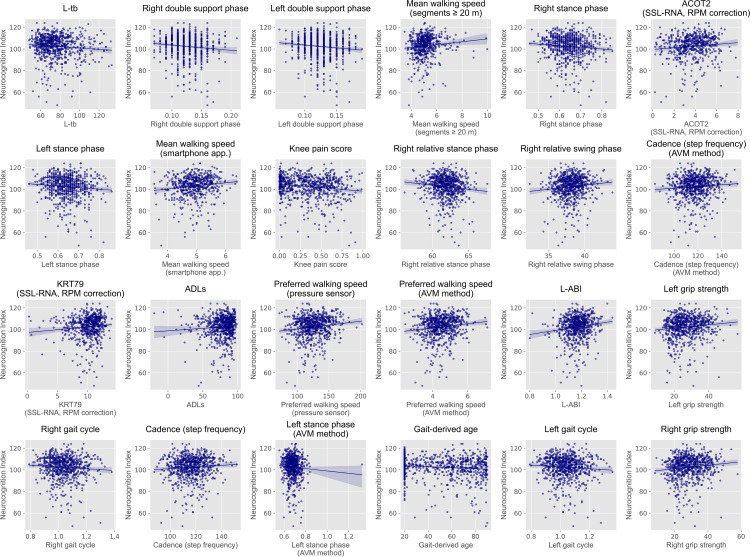
Scatter plots of NCI scores against the real type variables identified in partial correlation analysis. Each plot shows the relationship between NCI scores and the real type variables that exhibited partial correlations with cognitive function after adjustment for sex, age, and years of education (*q* < 0.1). Lines represent linear regression fits with 95% confidence intervals. NCI, Neurocognition Index.

**Fig 3:**
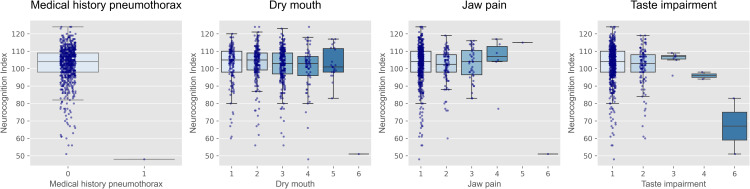
Box plots of NCI scores across the categorical type variables identified in ANCOVA. Each panel shows the distribution of NCI scores according to the categorical type variables that exhibited associations with cognitive function (*q* < 0.1) in the ANCOVA after adjustment for sex, age, and years of education. Variables include medical history of pneumothorax from the medical history questionnaire and oral conditions from the oral hygiene questionnaire. Boxes represent interquartile ranges, horizontal lines indicate medians, and dots represent individual participants. NCI, Neurocognition Index; ANCOVA, analysis of covariance.

The results of the association analyses are presented in Tables 2 and 3 and Figs 2 and 3. From the partial correlation analysis of the real type variables, many gait related variables measured using a sheet-type pressure sensor or a smartphone application were associated with cognitive function. For example, increases in double support phase, stance phase, and gait cycle, as well as decreases in walking speed, cadence (step frequency, expressed as steps per minute), and swing phase, were associated with lower cognitive function. In addition, a higher knee pain score and a lower Activities of Daily Living (ADLs) score, derived from gait characteristics, were both associated with lower cognitive function. Overall, participants with slower walking speed and longer gait cycle tended to exhibit lower cognitive function.

**Table 2:** Associations between NCI scores and the real type variables.

Variables	Category Field	*n*	Partial *r*	95% CI	*p*-value	*q*-value (FDR)
L-tb	Vascular function	708	−0.148	[ -0.22, -0.08 ]	< 0.001	0.0614
Right double support phase	Walking characteristics	710	−0.141	[ -0.21, -0.07 ]	< 0.001	0.0614
Left double support phase	Walking characteristics	710	−0.140	[ -0.21, -0.07 ]	< 0.001	0.0614
Mean walking speed (segments ≥20 m)	Walking characteristics	566	0.140	[ 0.06, 0.22 ]	< 0.001	0.0837
Right stance phase	Walking characteristics	710	−0.138	[ -0.21, -0.06 ]	< 0.001	0.0614
ACOT2 (SSL-RNA, RPM correction)	Biomarker	549	0.137	[ 0.05, 0.22 ]	0.00138	0.0892
Left stance phase	Walking characteristics	710	−0.136	[ -0.21, -0.06 ]	< 0.001	0.0614
Mean walking speed (smartphone app.)	Walking characteristics	551	0.133	[ 0.05, 0.21 ]	0.00187	0.0906
Knee pain score	Walking characteristics	709	−0.131	[ -0.20, -0.06 ]	< 0.001	0.0830
Right relative stance phase	Walking characteristics	709	−0.128	[ -0.20, -0.05 ]	< 0.001	0.0837
Right relative swing phase	Walking characteristics	709	0.126	[ 0.05, 0.20 ]	< 0.001	0.0837
Cadence (step frequency) (AVM method)	Walking characteristics	701	0.125	[ 0.05, 0.20 ]	< 0.001	0.0837
KRT79 (SSL-RNA, RPM correction)	Biomarker	614	0.125	[ 0.05, 0.20 ]	0.00201	0.0906
ADLs	Walking characteristics	710	0.124	[ 0.05, 0.20 ]	< 0.001	0.0837
Preferred walking speed (pressure sensor)	Walking characteristics	710	0.124	[ 0.05, 0.20 ]	< 0.001	0.0837
Preferred walking speed (AVM method)	Walking characteristics	701	0.123	[ 0.05, 0.20 ]	0.00109	0.0868
L-ABI	Vascular function	708	0.121	[ 0.05, 0.19 ]	0.00127	0.0892
Left grip strength	Motor function	710	0.120	[ 0.05, 0.19 ]	0.00137	0.0892
Right gait cycle	Walking characteristics	709	−0.119	[ -0.19, -0.05 ]	0.00159	0.0906
Cadence (step frequency)	Walking characteristics	709	0.118	[ 0.04, 0.19 ]	0.00165	0.0906
Left stance phase (AVM method)	Walking characteristics	701	−0.118	[ -0.19, -0.04 ]	0.00177	0.0906
Gait-derived age	Walking characteristics	710	−0.118	[ -0.19, -0.04 ]	0.00174	0.0906
Left gait cycle	Walking characteristics	709	−0.117	[ -0.19, -0.04 ]	0.00193	0.0906
Right grip strength	Motor function	709	0.115	[ 0.04, 0.19 ]	0.00220	0.0948

This table lists the real type variables that exhibited associations with cognitive function after adjustment for sex, age, and years of education. The variables are sorted by the absolute value of the partial correlation coefficient. The *p*-values were adjusted for multiple testing using FDR method and are reported as *q*-values. L-tb corresponds to the time from the first component of the second heart sound to the dicrotic notch of the left brachial pulse wave. NCI, Neurocognition Index; CI, confidence interval; FDR, false discovery rate; SSL-RNA, RNA in skin surface lipid; RPM, reads per million; AVM, around view monitor; ADLs, Activities of Daily Living; L-ABI, left ankle–brachial index. (See the details in S1 Table.)

**Table 3:** Associations between NCI scores and the categorical type variables.

Variables	Category Field	*n*	df	Partial $\eta_{p}^{2}$	F-value	*p*-value	*q*-value (FDR)
Medical history pneumothorax	Medical history / medication	710	1	0.0380	27.81	< 0.001	< 0.001
Dry mouth	Oral cavity	710	5	0.0334	4.85	< 0.001	0.0390
Jaw pain	Oral cavity	710	5	0.0300	4.34	< 0.001	0.0721
Taste impairment	Oral cavity	710	4	0.0299	5.40	< 0.001	0.0390

This table lists the categorical type variables that exhibited associations with cognitive function in the ANCOVA after adjustment for sex, age, and years of education. Variables are sorted by the partial $\eta_{p}^{2}$ values. The *p*-values were adjusted for multiple testing using FDR method and are reported as *q-*values. “Dry mouth”, “Jaw pain”, and “Taste impairment” were assessed using the oral hygiene questionnaire. NCI, Neurocognition Index; ANCOVA, analysis of covariance; df, degrees of freedom; FDR, false discovery rate. (See the details in S2 Table.)

Among vascular function related variables, a decrease in L-ABI, indicating a higher degree of vascular stenosis in the left leg, was associated with lower cognitive function. In addition, among motor function related variables, lower grip strength in both hands was associated with lower cognitive function. Furthermore, relationships were observed between cognitive function and several other factors, including L-tb (a waveform-derived left brachial pulse wave parameter used in CAVI calculation) and RNA expression levels in skin surface lipids.

From the ANCOVA results for the categorical variables, several oral health related variables were identified. Specifically, increased oral dryness and decreased sense of taste were associated with lower NCI scores, whereas increased jaw pain was associated with higher NCI scores. Additionally, a relationship was also observed between cognitive function and a medical history of pneumothorax. No variables with positive and ordered categorical types passed the exploratory FDR threshold (*q* < 0.1).

## Discussion

### Principal findings and interpretation

In this study, we conducted a comprehensive statistical analysis of a dataset comprising anthropometric characteristics, body composition, and lifestyle habits of Japanese adults aged 40 years or older, with the aim of exploring factors associated with cognitive function. After adjusting for sex, age, and years of education, we conducted partial correlation analysis and ANCOVA, which revealed that variables related primarily to gait characteristics, vascular function, motor function, and oral conditions were associated with cognitive function. By contrast, no associations involving common biochemical markers such as blood components passed the exploratory FDR threshold. Given the cross-sectional and exploratory nature of this study, these results do not allow causal inference, and lifestyle related indicators should be examined in future longitudinal studies.

### Interpretation of identified associations

Our finding that quantitative gait characteristics are associated with cognitive function is consistent with previous studies [64,65]. Gait is a complex motor activity that relies on multiple cognitive domains, including attention, planning, visuospatial processing, and motor control [66]. The observed associations suggest that quantitative gait characteristics may be candidate correlates of cognitive function. Daily gait monitoring with smartphones or wearable devices may support longitudinal studies examining changes in cognitive function among adults aged 40 years or older.

ABI is a well-established indicator for assessing the degree of vascular stenosis or occlusion in the lower limbs due to atherosclerosis, and it is widely employed as a non-invasive screening tool for peripheral artery disease [67]. Our results, showing that reduced ABI values (reflecting greater vascular stenosis) were associated with lower cognitive function, are consistent with previous report [68]. Vascular narrowing may be related to chronic cerebral hypoperfusion, which has been associated with reduced oxygen supply, neurodegeneration, and impaired clearance of amyloid-β [69,70]. These changes may be associated with cognitive function.

L-tb is a waveform-derived time parameter measured by the vascular screening system and used to calculate CAVI, an index of arteriosclerosis [71]. It may be influenced by pulse wave reflection and peripheral vascular resistance. An association between the pulse wave parameter and cognitive function is consistent with prior studies reporting that higher pulse wave velocity is associated with lower cognitive function [72,73]. Pulse wave velocity is an established indicator of arteriosclerosis [74]. This finding is consistent with the hypothesis that vascular factors may be related to cognitive function, as also suggested by the association observed for ABI.

Handgrip strength is a well-recognized biomarker of physical function and aging, and its association with cognitive function has been reported in many studies [75–77]. Several hypotheses have been proposed to account for this association. For instance, muscle weakness and cognitive impairment may share common underlying factors, such as high levels of inflammatory markers and reduced sex steroid levels [78,79]. Muscle strength may also reflect aspects of central nervous system integrity, and decreased muscle strength may be consistent with an overall reduction in neural processing activity that could be accompanied by lower performance across multiple cognitive domains [80,81]. However, due to the cross sectional nature of the data, the direction of the association cannot be determined, and reverse causation and residual confounding cannot be ruled out. Although the present dataset included several body composition variables that reflect total muscle mass, none of these variables passed the exploratory FDR threshold. This finding may suggest that muscle mass does not necessarily reflect muscle strength, whereas grip strength which requires fine and coordinated movements of the hand and forearm may better capture neuromuscular efficiency. This difference may partly account for the stronger observed association between grip strength and cognitive function.

The relationship between oral conditions and cognitive function has been increasingly reported in recent years [82–84]. Tooth loss, for example, has been associated with reduced masticatory function and linked to lower hippocampal neuron numbers, reduced central nervous system activity, and smaller brain volume [85]. Salivary hyposecretion has been related to xerostomia (dry mouth), taste disorders, and periodontal diseases [86,87]. In a rat model study, periodontitis was reported to elevate inflammatory cytokine levels in the brain, which may be relevant to hypotheses regarding the pathogenesis of dementia [88]. Higher self-reported jaw pain was associated with higher NCI scores, which is counter to prior reports linking reduced masticatory function to lower cognitive function [89]. However, the direction of this association cannot be determined from the cross sectional design. Because jaw pain may capture heterogeneous conditions and may be subject to residual confounding bias, this finding should be interpreted cautiously and reassessed in independent longitudinal studies.

### Exploratory findings

We also observed associations between cognitive function and a medical history of pneumothorax, as well as SSL-RNA markers (ACOT2 and KRT79). However, these findings should be interpreted with great caution. These associations are not well supported by prior research. Given the exploratory nature of the study and the large number of tests performed, we cannot rule out the possibility that they reflect chance findings due to multiple comparisons. In particular, the apparent strong association between a medical history of pneumothorax and cognitive function is likely a statistical artifact, as only a small number of participant reported such a history. For the SSL-RNA markers, the *q*-values were close to the exploratory FDR threshold (0.089–0.091).

### Methodological considerations and robustness checks

In this study, we used the NCI score, a composite measure, as our primary indicator of cognitive function, rather than scores for individual cognitive domains. We observed moderate correlations among CNSVS subdomain scores (S1 Fig) and anticipated that testing multiple domains separately would increase the burden of multiple comparisons and complicate interpretation. We judged that using a single composite measure would be the most statistically appropriate approach.

We applied FDR correction separately within each data type. We consider this appropriate for the categorical variables because they require statistical analysis methods different from those used for other data types. Moreover, if data types whose statistical properties and dependency structures differ greatly are pooled for a single correction, the number of hypotheses of one type may unduly affect the statistical power for other types. To avoid this, we conducted FDR correction also separately within the real, positive, and ordered categorical types, which we consider to have similar statistical characteristics. To evaluate the validity of this approach, we performed a global FDR correction as a sensitivity analysis, applying it to the partial correlation analyses on the combined set of real, positive, and ordered categorical variables. The results are presented in the supplementary materials (S3 Table). Although the number of identified variables decreased and only a subset of variables related to vascular function and gait characteristics was extracted, we confirmed that the conclusions were not inconsistent.

We set the *q*‑value threshold at 0.1 as a pragmatic criterion for this exploratory and hypothesis‑generating analysis. Using a stricter cutoff of *q* < 0.05, only three associations in the same analysis passed the threshold, namely a medical history of pneumothorax and two oral health related variables (S4 Table). As noted earlier, the association with a medical history of pneumothorax is likely a chance finding (see Exploratory findings). By contrast, the oral health related variables continued to pass the stricter threshold, which may indicate comparatively more consistent associations with cognitive function.

To handle missing data, we used complete‑case analysis. Complete‑case analysis can introduce bias when missingness depends on cognitive function or health status. Of the 28 variables identified in this study, 24 had missing rates below 2%, suggesting that adopting complete-case analysis was unlikely to introduce bias. In contrast, four variables related to walking speed and SSL-RNA had missing rates exceeding 10%. To evaluate potential bias for these variables, we compared key demographics (sex, age, and years of education) and the NCI score between complete‑case and incomplete‑case groups using chi‑square tests and Welch’s *t* tests with α=0.05 (S5 Table). We observed between-group differences for several variables, which are consistent with missingness depending on observed variables (missing at random, MAR). We therefore conducted sensitivity analyses using multiple imputation by chained equations (MICE), analyzed imputed datasets with the same pipeline, and pooled estimates using Rubin’s rules. As a result, although a small number of variables no longer passed the exploratory FDR threshold after imputation, results from the imputed datasets were largely consistent with the original complete‑case analysis and our conclusions remained unchanged (S6 Table).

While some variables passed the exploratory FDR threshold (*q* < 0.1), many showed small effect sizes (e.g., partial correlation coefficients |r|≈0.11-0.15). Therefore, these individual indicators are unlikely to be clinically actionable as standalone screening markers. However, we observed a consistent pattern of small associations across multiple variables within specific physiological domains, such as gait, vascular function, grip strength, and oral health. This domain-level consistency suggests that multivariable or composite indices integrating several measures may be more informative, although their clinical utility should be evaluated in independent cohorts and prospective studies.

### Limitations

This study has several limitations. First, there is a potential for bias arising from the exclusion of participants with missing data. Most variables had very little missing data, but four variables (walking speed and SSL-RNA) had more than 10% missing data. Missingness for these variables may have been related to participant characteristics (e.g., ability to complete device-based measurements, availability of sufficient sebum for analysis), which could introduce systematic bias. Although our main models adjusted for sex, age, and years of education, selection bias may still occur if missingness depends on cognitive function or other unmeasured factors. Sensitivity analyses using multiple imputation were broadly consistent with the primary analysis, but a few associations became weaker, so some bias may remain. In addition, because we initially analyzed only complete cases, we may have favored variables with less missing data, meaning that associations for variables with more missing data could have been missed or underestimated.

Second, the observed associations do not establish causality, and the results should be interpreted with caution. The discussion of potential mechanisms examines the biological plausibility of the observed associations, without implying causality, and is intended to generate hypotheses for future prospective cohort studies and intervention trials.

Third, there are limitations to the generalizability of the results. Participants were primarily middle-aged and older adults living in urban areas of Japan, and they were relatively healthy and volunteered to take part in the study. Therefore, these findings may not directly apply to groups with different backgrounds, for example rural residents or groups with specific health conditions, or to populations in other countries or with other ethnic backgrounds. In particular, the measures found to be associated in this study, such as gait and oral health, can be strongly influenced by a country’s culture, living environment, and healthcare system. For example, factors such as the availability of public transportation, dietary habits, and access to dental checkups could alter the strength of the associations between these indicators and cognitive function. To assess the generalizability of these findings, future research targeting populations with more diverse backgrounds is needed.

Considering these limitations, further studies are warranted to elucidate the mechanisms and potential causal relationships underlying the findings of this study.

## Conclusion

In this study, we analyzed data on 710 Japanese adults aged 40 years or older from a multi-parameter cross-sectional study conducted in 2021–2022. Using partial correlation analysis and analysis of covariance adjusted for sex, age, and years of education, we examined associations between cognitive function assessed using CNS Vital Signs and approximately 1,800 variables encompassing physical characteristics, body composition, and lifestyle habits. We identified 28 variables related to gait, vascular function, grip strength, and oral condition that were associated with cognitive function. These exploratory associations, observed in this relatively healthy and primarily urban Japanese cohort, should not be interpreted as evidence of causality and require confirmation in independent and diverse populations, ideally using longitudinal and interventional study designs.

## Supporting information

S1 Fig Correlations among CNSVS subdomain scores and the NCI score. This supplementary figure shows Spearman rank correlation coefficients between CNSVS subdomain scores and the NCI score. The color scale indicates the correlation coefficient, and numeric values are shown in each cell.
(TIF)

S1 Table Detailed descriptions of the real type variables associated with cognitive function. This supplementary table provides detailed definitions and measurement descriptions for the real type variables that showed partial correlations with NCI scores after adjustment for sex, age, and years of education.
(DOCX)

S2 Table Detailed descriptions of the categorical type variables associated with cognitive function. This supplementary table provides detailed definitions and response options for the categorical type variables that showed associations with NCI scores in ANCOVA after adjustment for sex, age, and years of education.
(DOCX)

S3 Table Sensitivity analysis using a global FDR correction across the real, positive, and ordered categorical variables. This supplementary table provides the results of partial correlation analyses between the NCI score and lifestyle/physiological variables when the FDR is controlled globally across the combined set of real, positive, and ordered categorical variables. Partial Spearman correlations were adjusted for sex, age, and years of education. The *p*-values were corrected using the Benjamini-Hochberg procedure and variables with *q* < 0.1 are listed.
(DOCX)

S4 Table Associations that passed a stricter multiple-testing threshold. This supplementary table summarizes variables that passed the more conservative FDR threshold of *q* < 0.05 in the prespecified analyses.
(DOCX)

S5 TableComparison of complete-case vs. incomplete-case participants for variables with greater than 10% missing rates.This supplementary table compares the key characteristics between complete-case and incomplete-case groups for the four variables that exhibited missing rates >10% (variables related to walking speed and SSL-RNA). Group differences were assessed for sex (chi square tests) and for age, years of education, and NCI score (Welch’s *t* tests), with α=0.05.(DOCX)

S6 Table Multiple imputation sensitivity analysis for the variables with greater than 10% missing rates. This supplementary table provides the results from sensitivity analysis after multiple imputation by MICE for the four variables with > 10% missing rates (walking speed and SSL-RNA). Imputation models included sex, age, BMI, years of education, smoking status, drinking frequency, exercise frequency, NCI score, and the three variables most strongly correlated with each target variable. We generated 30 imputed datasets after 20 burn-in iterations and conducted the prespecified association analysis (partial correlations for the real, positive, and ordered categorical variables). Parameter estimates and their variances were pooled using Rubin’s rules and applied FDR correction using Benjamini-Hochberg procedure within data type. Variables with *q* < 0.1 are reported.
(DOCX)

S1 Code Analysis code for the main analyses. This notebook (.ipynb) contains the code used to reproduce the main analyses reported in the manuscript, including data preprocessing, statistical analyses, and generation of the main figures and tables.
(IPYNB)

S2 Code Analysis code for the supplementary analyses. This notebook (.ipynb) contains the code used to reproduce the supplementary analyses reported in the Supporting Information, including sensitivity analyses, robustness checks, and generation of the supplementary figure and tables.
(IPYNB)
