# Supplementary figures and images for: Associations between cognitive function and lifestyle factors in healthy Japanese middle-aged and older adults: A cross-sectional study
Source: PLoS One. 2026 May 4;21(5):e0348439. doi: 10.1371/journal.pone.0348439 (PMC13138663; doi:10.1371/journal.pone.0348439)

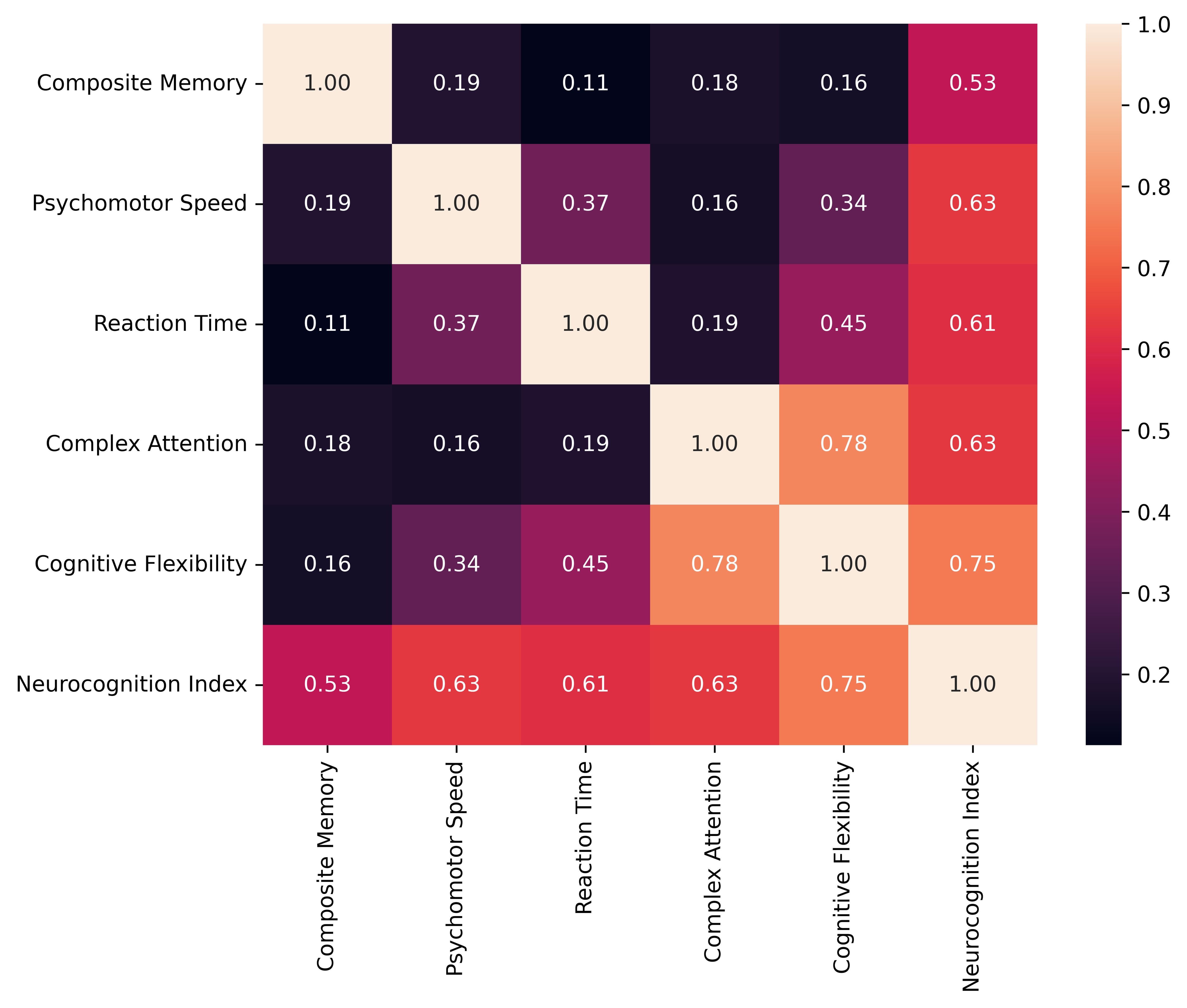

Supplement: S1 Fig — This supplementary figure shows Spearman rank correlation coefficients between CNSVS subdomain scores and the NCI score. The color scale indicates the correlation coefficient, and numeric values are shown in each cell. (TIF) [file pone.0348439.s001.tif]
