# Supplementary material for: Associations between cognitive function and lifestyle factors in healthy Japanese middle-aged and older adults: A cross-sectional study
Source: PLoS One. 2026 May 4;21(5):e0348439. doi: 10.1371/journal.pone.0348439 (PMC13138663; doi:10.1371/journal.pone.0348439)
Supplement: S2 Table — This supplementary table provides detailed definitions and response options for the categorical type variables that showed associations with NCI scores in ANCOVA after adjustment for sex, age, and years of education. (DOCX) [file pone.0348439.s003.docx]

**S2 Table. Detailed descriptions of the categorical type variables associated with cognitive function.** This supplementary table provides detailed definitions, response options, and counts for the categorical type variables that showed associations with Neurocognition Index (NCI) scores in the analysis of covariance (ANCOVA) after adjustment for sex, age, and years of education.

Each variable was assigned to one of the predefined category field (e.g., Oral cavity) based on its characteristics. Variables were derived from the medical history questionnaire and the oral hygiene questionnaire administered in the cross-sectional study.

| **Variable name** | **Category field** | **Description** | **Response options (value labels)** | **Count** |
| --- | --- | --- | --- | --- |
| **Medical history pneumothorax** | Medical history / medication | Self-reported history of pneumothorax. Participants were asked whether they had ever been diagnosed with pneumothorax. | 0 = No (no history); 1 = Yes (history present) | 0 = 709; 1 = 1 |
| **Dry mouth** | Oral cavity | Frequency of perceived oral dryness during the past 2–3 months was assessed using the following question:  “Do you feel that your mouth is dry?” | 1 = Never; 2 = Rarely; 3 = Sometimes; 4 = Often; 5 = Always; 6 = Do not know | 1 = 146; 2 = 206; 3 = 269; 4 = 65; 5 = 23; 6 = 1 |
| **Jaw pain** | Oral cavity | Frequency of pain in the jaw area during the past 2–3 months was assessed using the following question:  “Do you experience pain in your jaw?” | 1 = Never; 2 = Rarely; 3 = Sometimes; 4 = Often; 5 = Always; 6 = Do not know | 1 = 557; 2 = 102; 3 = 43; 4 = 6; 5 = 1; 6 = 1 |
| **Taste impairment** | Oral cavity | Degree of difficulty perceiving taste during the past 2–3 months was assessed using the following question:  “Do you have difficulty perceiving taste?” | 1 = Never; 2 = Rarely; 3 = Sometimes; 4 = Often or Always; 6 = Do not know | 1 = 586; 2 = 113; 3 = 7; 4 = 2; 6 = 2 |
