# Supplementary material for: Associations between cognitive function and lifestyle factors in healthy Japanese middle-aged and older adults: A cross-sectional study
Source: PLoS One. 2026 May 4;21(5):e0348439. doi: 10.1371/journal.pone.0348439 (PMC13138663; doi:10.1371/journal.pone.0348439)
Supplement: S3 Table — This supplementary table provides the results of partial correlation analyses between the NCI score and lifestyle/physiological variables when the FDR is controlled globally across the combined set of real, positive, and ordered categorical variables. Partial Spearman correlations were adjusted for sex, age, and years of education. The p-values were corrected using the Benjamini-Hochberg procedure and variables with q < 0.1 are listed. (DOCX) [file pone.0348439.s004.docx]

**S3 Table. Sensitivity analysis using a global FDR correction across the real, positive, and ordered categorical variables.**

This supplementary table provides the results of partial correlation analyses between the NCI score and lifestyle/physiological variables when the FDR is controlled globally across the combined set of real, positive, and ordered categorical variables. Partial Spearman correlations were adjusted for sex, age, and years of education. The *p*-values were corrected using the Benjamini-Hochberg procedure and variables with *q* < 0.1 are listed. Abbreviations: NCI, Neurocognition Index; FDR, false discovery rate; CI, confidence interval.

| **Variables** | **Category Field** | ***n*** | **Partial r** | **95% CI** | ***p*-value** | ***q*-value (FDR)** |
| --- | --- | --- | --- | --- | --- | --- |
| **L-tb** | Vascular function | 708 | -0.148 | [-0.22, -0.08] | < 0.001 | 0.0761 |
| **Right double support phase** | Walking characteristics | 710 | -0.141 | [-0.21, -0.07] | < 0.001 | 0.0761 |
| **Left double support phase** | Walking characteristics | 710 | -0.140 | [-0.21, -0.07] | < 0.001 | 0.0761 |
| **Right stance phase** | Walking characteristics | 710 | -0.138 | [-0.21, -0.06] | < 0.001 | 0.0761 |
| **Left stance phase** | Walking characteristics | 710 | -0.136 | [-0.21, -0.06] | < 0.001 | 0.0761 |
