# Supplementary material for: Associations between cognitive function and lifestyle factors in healthy Japanese middle-aged and older adults: A cross-sectional study
Source: PLoS One. 2026 May 4;21(5):e0348439. doi: 10.1371/journal.pone.0348439 (PMC13138663; doi:10.1371/journal.pone.0348439)
Supplement: S4 Table — This supplementary table summarizes variables that passed the more conservative FDR threshold of q < 0.05 in the prespecified analyses. (DOCX) [file pone.0348439.s005.docx]

**S4 Table. Associations that passed a stricter multiple-testing threshold.**

This supplementary table summarizes variables that passed the more conservative FDR threshold of *q* < 0.05 in the prespecified analyses (partial correlation for the real, positive, or ordered categorical variables and ANCOVA for the categorical variables), each corrected within its data type. Under this stricter cutoff, only three categorical variables remained below the FDR threshold: a medical history of pneumothorax and two oral health related variables from the oral hygiene questionnaire. Results are reported in the same format as the main tables. Abbreviations: FDR, false discovery rate; ANCOVA, analysis of covariance.

| **Variables** | **Category Field** | ***n*** | **df** | **Partial eta squared** | **F-value** | ***p*-value** | ***q*-value (FDR)** |
| --- | --- | --- | --- | --- | --- | --- | --- |
| **Medical history pneumothorax** | Medical history / medication | 710 | 1 | 0.0380 | 27.81 | < 0.001 | < 0.001 |
| **Dry mouth** | Oral cavity | 710 | 5 | 0.0334 | 4.85 | < 0.001 | 0.0390 |
| **Taste impairment** | Oral cavity | 710 | 4 | 0.0299 | 5.40 | < 0.001 | 0.0390 |
