# Supplementary material for: Associations between cognitive function and lifestyle factors in healthy Japanese middle-aged and older adults: A cross-sectional study
Source: PLoS One. 2026 May 4;21(5):e0348439. doi: 10.1371/journal.pone.0348439 (PMC13138663; doi:10.1371/journal.pone.0348439)
Supplement: S5 Table — This supplementary table compares the key characteristics between complete-case and incomplete-case groups for the four variables that exhibited missing rates >10% (variables related to walking speed and SSL-RNA). Group differences were assessed for sex (chi square tests) and for age, years of education, and NCI score (Welch’s t tests), with α=0.05. (DOCX) [file pone.0348439.s006.docx]

**S5 Table. Comparison of complete-case vs. incomplete-case participants for variables with greater than 10% missing rates.**

This supplementary table compares the key characteristics between complete-case and incomplete-case groups for the four variables that exhibited missing rates > 10% (variables related to walking speed and SSL-RNA). Group differences were assessed for sex (chi square test) and for age, years of education, and NCI score (Welch’s *t* test), with α = 0.05. Abbreviations: NCI, Neurocognition Index; SSL-RNA, RNA in skin surface lipids; RPM, reads per million.

**Mean walking speed (segments ≥ 20 m)**

|  | **Complete-case** | **Incomplete-case** | ***p*-value** |
| --- | --- | --- | --- |
| **Sample number** | 566 | 144 | - |
| **Sex** | - | - | 0.7490 |
| Male count | 290 | 71 | - |
| Female count | 276 | 73 | - |
| **Mean age** | 58.6 | 62.2 | < 0.001 |
| **Mean year of education** | 15.0 | 15.1 | 0.5328 |
| **Mean NCI score** | 102.8 | 100.6 | 0.0471 |

**ACOT2 (SSL-RNA, RPM correction)**

|  | **Complete-case** | **Incomplete-case** | ***p*-value** |
| --- | --- | --- | --- |
| **Sample number** | 549 | 161 | - |
| **Sex** | - | - | < 0.001 |
| Male count | 227 | 134 | - |
| Female count | 322 | 27 | - |
| **Mean age** | 58.4 | 62.3 | <0.001 |
| **Mean year of education** | 15.1 | 14.4 | <0.001 |
| **Mean NCI score** | 102.5 | 102.0 | 0.5714 |

**Mean walking speed (smartphone app.)**

|  | **Complete-case** | **Incomplete-case** | ***p*-value** |
| --- | --- | --- | --- |
| **Sample number** | 551 | 159 | - |
| **Sex** | - | - | 0.6324 |
| Male count | 277 | 84 | - |
| Female count | 274 | 75 | - |
| **Mean age** | 58.3 | 62.7 | < 0.001 |
| **Mean year of education** | 15.0 | 15.0 | 0.9472 |
| **Mean NCI score** | 102.9 | 100.6 | 0.0282 |

**KRT79 (SSL-RNA, RPM correction)**

|  | **Complete-case** | **Incomplete-case** | ***p*-value** |
| --- | --- | --- | --- |
| **Sample number** | 614 | 96 | - |
| **Sex** | - | - | < 0.001 |
| Male count | 282 | 79 | - |
| Female count | 332 | 17 | - |
| **Mean age** | 58.9 | 62.2 | 0.0069 |
| **Mean year of education** | 15.1 | 14.4 | 0.0024 |
| **Mean NCI score** | 102.5 | 101.4 | 0.3059 |
