# Supplementary material for: Associations between cognitive function and lifestyle factors in healthy Japanese middle-aged and older adults: A cross-sectional study
Source: PLoS One. 2026 May 4;21(5):e0348439. doi: 10.1371/journal.pone.0348439 (PMC13138663; doi:10.1371/journal.pone.0348439)
Supplement: S6 Table — This supplementary table provides the results from sensitivity analysis after multiple imputation by MICE for the four variables with > 10% missing rates (walking speed and SSL-RNA). Imputation models included sex, age, BMI, years of education, smoking status, drinking frequency, exercise frequency, NCI score, and the three variables most strongly correlated with each target variable. We generated 30 imputed datasets after 20 burn-in iterations and conducted the prespecified association analysis (partial correlations for the real, positive, and ordered categorical variables). Parameter estimates and their variances were pooled using Rubin’s rules and applied FDR correction using Benjamini-Hochberg procedure within data type. Variables with q < 0.1 are reported. (DOCX) [file pone.0348439.s007.docx]

**S6 Table. Multiple imputation sensitivity analysis for the variables with greater than 10% missing rates.**

This supplementary table provides the results from sensitivity analysis after multiple imputation by MICE for the four variables with > 10 % missing rates (walking speed and SSL-RNA). Imputation models included sex, age, BMI, years of education, smoking status, drinking frequency, exercise frequency, NCI score, and the three variables most strongly correlated with each target variable. We generated 30 imputed datasets after 20 burn-in iterations and conducted the prespecified association analysis (partial correlations for the real, positive, and ordered categorical variables). Parameter estimates and their variances were pooled using Rubin’s rules and applied FDR correction using Benjamini-Hochberg procedure within data types. Variables with *q* < 0.10 are reported. Abbreviations: MICE, multiple imputation by chained equations; SSL-RNA, RNA in skin surface lipids; BMI, body mass index; NCI, Neurocognition Index; FDR, false discovery rate.

| **Variables** | **Category Field** | ***n*** | **Partial r** | **95% CI** | ***p*-value** | ***q*-value (FDR)** |
| --- | --- | --- | --- | --- | --- | --- |
| **L-tb** | Vascular function | 708 | -0.148 | [-0.22, -0.08] | < 0.001 | 0.0614 |
| **Right double support phase** | Walking characteristics | 710 | -0.141 | [-0.21, -0.07] | < 0.001 | 0.0614 |
| **Left double support phase** | Walking characteristics | 710 | -0.140 | [-0.21, -0.07] | < 0.001 | 0.0614 |
| **Right stance phase** | Walking characteristics | 710 | -0.138 | [-0.21, -0.06] | < 0.001 | 0.0614 |
| **Left stance phase** | Walking characteristics | 710 | -0.136 | [-0.21, -0.06] | < 0.001 | 0.0614 |
| **Mean walking speed (smartphone app.)** | Walking characteristics | 710 | 0.134 | [0.06, 0.21] | < 0.001 | 0.0821 |
| **ACOT2 (SSL-RNA, RPM correction)** | Biomarker | 710 | 0.133 | [0.05, 0.21] | 0.00111 | 0.0821 |
| **Knee pain score** | Walking characteristics | 709 | -0.131 | [-0.2, -0.06] | < 0.001 | 0.0821 |
| **Right relative stance phase** | Walking characteristics | 709 | -0.128 | [-0.2, -0.05] | < 0.001 | 0.0821 |
| **Right relative swing phase** | Walking characteristics | 709 | 0.126 | [0.05, 0.2] | < 0.001 | 0.0821 |
| **Cadence (step frequency) (AVM method)** | Walking characteristics | 701 | 0.125 | [0.05, 0.2] | < 0.001 | 0.0821 |
| **ADLs** | Walking characteristics | 710 | 0.124 | [0.05, 0.2] | < 0.001 | 0.0821 |
| **Preferred walking speed (pressure sensor)** | Walking characteristics | 710 | 0.124 | [0.05, 0.2] | < 0.001 | 0.0821 |
| **Preferred walking speed (AVM method)** | Walking characteristics | 701 | 0.123 | [0.05, 0.2] | 0.00109 | 0.0821 |
| **L-ABI** | Vascular function | 708 | 0.121 | [0.05, 0.19] | 0.00127 | 0.0874 |
| **Left grip strength** | Motor function | 710 | 0.120 | [0.05, 0.19] | 0.00137 | 0.0886 |
| **Right gait cycle** | Walking characteristics | 709 | -0.119 | [-0.19, -0.05] | 0.00159 | 0.0918 |
| **Cadence (step frequency)** | Walking characteristics | 709 | 0.118 | [0.04, 0.19] | 0.00165 | 0.0918 |
| **Left stance phase (AVM method)** | Walking characteristics | 701 | -0.118 | [-0.19, -0.04] | 0.00177 | 0.0918 |
| **Gait-derived age** | Walking characteristics | 710 | -0.118 | [-0.19, -0.04] | 0.00174 | 0.0918 |
| **Left gait cycle** | Walking characteristics | 709 | -0.117 | [-0.19, -0.04] | 0.00193 | 0.0949 |
